# Moving the Shh Source over Time: What Impact on Neural Cell Diversification in the Developing Spinal Cord?

**DOI:** 10.3390/jdb5020004

**Published:** 2017-04-12

**Authors:** Cathy Danesin, Cathy Soula

**Affiliations:** Centre de Biologie du Développement (CBD) CNRS/UPS, Centre de Biologie Intégrative (CBI), Université de Toulouse, 31520 Toulouse, France

**Keywords:** Sonic Hedgehog, neural tube patterning, notochord, medial floor plate, lateral floor plate, neurons, glial cells, amniotes, zebrafish

## Abstract

A substantial amount of data has highlighted the crucial influence of Shh signalling on the generation of diverse classes of neurons and glial cells throughout the developing central nervous system. A critical step leading to this diversity is the establishment of distinct neural progenitor cell domains during the process of pattern formation. The forming spinal cord, in particular, has served as an excellent model to unravel how progenitor cells respond to Shh to produce the appropriate pattern. In recent years, considerable advances have been made in our understanding of important parameters that control the temporal and spatial interpretation of the morphogen signal at the level of Shh-receiving progenitor cells. Although less studied, the identity and position of Shh source cells also undergo significant changes over time, raising the question of how moving the Shh source contributes to cell diversification in response to the morphogen. Here, we focus on the dynamics of Shh-producing cells and discuss specific roles for these time-variant Shh sources with regard to the temporal events occurring in the receiving field.

## 1. Introduction

**Figure 1:**
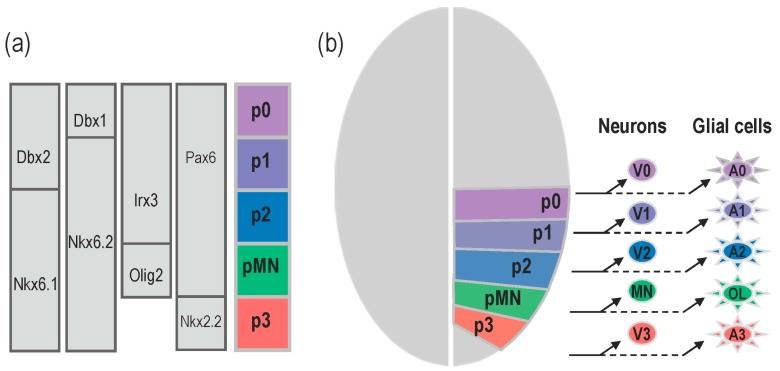
Domain organisation of ventral neural progenitor cells controls generation of distinct neuronal and glial cell subtypes. (**a**) Five progenitor domains, p3, pMN, p2, p1 and p0, are established in response to Shh along the ventral to dorsal axis. Progenitor cells in each domain are characterised by the combinatorial expression of transcription factors indicated on the left. (**b**) Each progenitor pool sequentially generates a specific sub-type of neurons and glial cells. Progenitor cells of the p0, p2 and p3 domains first produce three distinct types of ventral interneurons (V0–V3) and, later on, change their fate to generate ventral astrocytes (A0–A3). Progenitor cells of the pMN domain characterised by Olig2 expression first generate motor neurons (MN) and switches to production of oligodendroglial cells (OL).

The functional complexity of the vertebrate central nervous system is reflected by the large variety of neurons that form complex processing networks but also by the diversity of their indispensable glial cell partners, mainly composed of astrocytes and oligodendrocytes. Generation of this large array of distinct neural cell types is initiated at early stages of embryonic development and depends on the influence of a limited number of signalling cues. Among them, the morphogen factor Sonic Hedgehog (Shh) appears central in governing the specification of a variety of neuronal and glial cell lineages. When and how Shh controls the identity and pattern of neural cell types is best understood in the ventral region of the forming spinal cord. In this tissue, Shh acts as a long-range morphogen to direct the pattern of neurogenesis by conferring positional information to neural progenitor cells. During the transformation of the neural plate into the neural tube, Shh triggers the elaboration of five discrete domains of progenitor cells, named p3, pMN, p2, p1, and p0, arrayed along the dorso-ventral axis [1,2]. Identities of progenitor domains are based on the combinatorial expression of a set of transcription factors and these specific combinatorial codes are necessary and sufficient to specify the neuronal subtypes that each domain generates [3,4]. The pMN progenitor domain gives rise to motor neurons (MNs), while the p3, p2, p1, and p0 progenitor cells generate V3, V2, V1, and V0 interneurons, respectively (Figure 1). After completion of neuronal production, a neuronal to glial switch operates in the ventral spinal cord and a similar principle of progenitor domain organization regulates the generation of distinct glial cell sub-types [5] (Figure 1). Distinct populations of astrocytes originate from the p3, p2, p1 and p0 domains [6,7,8] while progenitor cells of the pMN domain generate oligodendrocytes [9,10,11,12].

Many studies have been conducted to elucidate the complex process of patterning establishment in response to Shh and, although still incomplete, significant progress was made in understanding the fundamental basis of this process. In the presumptive spinal cord region, Shh is initially secreted from the notochord, a population of mesodermal cells, acting as an organizing centre for the overlying neural tissue [13,14]. In amniotes, Shh provided by the notochord induces the formation of a second centre of Shh production at the midline of the neural tube, named the floor plate [15,16,17]. Shh provided by these ventral axial structures spreads through the ventral neural tube and establishes a gradient of activity necessary for pattern formation [18,19,20]. This graded information is sensed by ventral progenitor cells that express Patched (Ptch1), the Shh receptor, and is transduced into the receiving cells by the transmembrane protein Smoothened (Smo), which in turn controls the downstream activation of Gli transcriptional effectors [2,21]. Progenitor cells then respond to the graded Shh signalling by regulating the expression of homeodomain and basic helix-loop-helix transcription factors that have been subdivided into two groups, termed class I and II proteins, on the basis of their mode of regulation by Shh signalling [22]. Class I genes, such as Irx3, and Pax6 are repressed by distinct thresholds of Shh activity, while class II genes, including Nkx6.1, Nkx2.2 and Olig2, are activated by graded Shh signalling (Figure 1). The combinatorial expression of class I and II proteins together with cross-repressive interactions between them contribute to establish boundaries of gene expression that divide the ventral neural tube into the five above-mentioned progenitor domains [2]. As an example, Nkx2.2, requiring high Shh concentrations to be induced, is expressed in the ventral-most progenitor cells of the p3 domain, whereas Olig2, induced by lower Shh concentrations, is expressed more dorsally, in the pMN domain [23]. However, Shh concentration is not the only level of regulation as duration of exposure to Shh also influences the response to the morphogen, with ventral identities requiring longer time of Shh exposure to be established [24,25]. Therefore, progenitor domains form sequentially and the ventral-most p3 domain emerges later than the dorsally located pMN domain [24,25,26,27,28,29]. Expression of Olig2 in the ventral neural tube is indeed initiated before that of Nkx2.2. The ventral-most cells further activate the high threshold Shh responsive gene Nkx2.2 [28,30,31] which is in turn responsible for the downregulation of Olig2 and, thereby, for the formation of the two non-overlapping domains. Accordingly, progressive formation of ventral progenitor domains is accompanied by a temporal increase in the amplitude of the Shh gradient [19,20]. However, the levels of Shh signalling in progenitor cells are also dynamic and do not strictly correlate Shh protein distribution [20,27,32]. This adaptive response, referred to as temporal adaptation, is known to rely on dynamics of downstream Gli activity as well as regulation of co-receptors or inhibitors of Shh signalling [20,26,27,32,33]. Feedback activities exerted by homeodomain proteins also modulate the strength of Shh signalling in the ventral-most progenitor cells [28]. Thus, production of the appropriate pattern results from a complex interplay between the threshold of the Shh signal and the temporal adaptive response of receiving cells that actively collaborate to interpret and refine the morphogen gradient.

After that time, and only once neurons have been generated, progenitor cells of the ventral spinal cord change their fate and start generating glial cells in a process called the neuroglial switch. This process has been particularly well studied for the oligodendrocyte lineage. Most oligodendrocytes of the spinal cord are known to originate from Olig2-expressing progenitor cells [34,35]. Therefore, in the ventral spinal cord, newly specified oligodendrocyte precursor cells are positioned in the pMN domain, which, just before the initiation of gliogenesis, generates MNs. Although originating from the same progenitor domain, MNs and oligodendroglial cells have been shown to arise from distinct progenitor cells in the zebrafish embryo [36]. In amniotes, whether MNs and oligodendrocytes originate from common or separate lineages in vivo remains to be established [5]. Strikingly, the decision to produce oligodendroglial cells, long after patterning establishment, still depends on Shh signalling activity [37,38,39,40]. A set of studies reported that this cell fate change relies on a temporal rise of Shh signalling activity that occurs immediately prior to the MN to oligodendroglial cell fate switch [38,41,42,43]. Accordingly, this late activation of Shh signalling results in a reorganization of the ventral progenitor domains. Expression of Nkx2.2 extends dorsally and starts overlapping with the pMN domain precisely where MN production stops in the ventral spinal cord [39,42,43,44,45,46]. This leads to the formation of a new domain, named the p* domain, populated by progenitor cells that co-express Olig2 and Nkx2.2. Strikingly, at that time, Nkx2.2 no longer represses Olig2 and co-expression of the two transcription factors is critical to drive p* progenitor cells towards the oligodendroglial fate [44]. Thus, the Shh signal must not only be provided until late developmental stages but also strengthened to allow full accomplishment of the neural differentiation repertoire in the ventral spinal cord.

**Figure 2:**
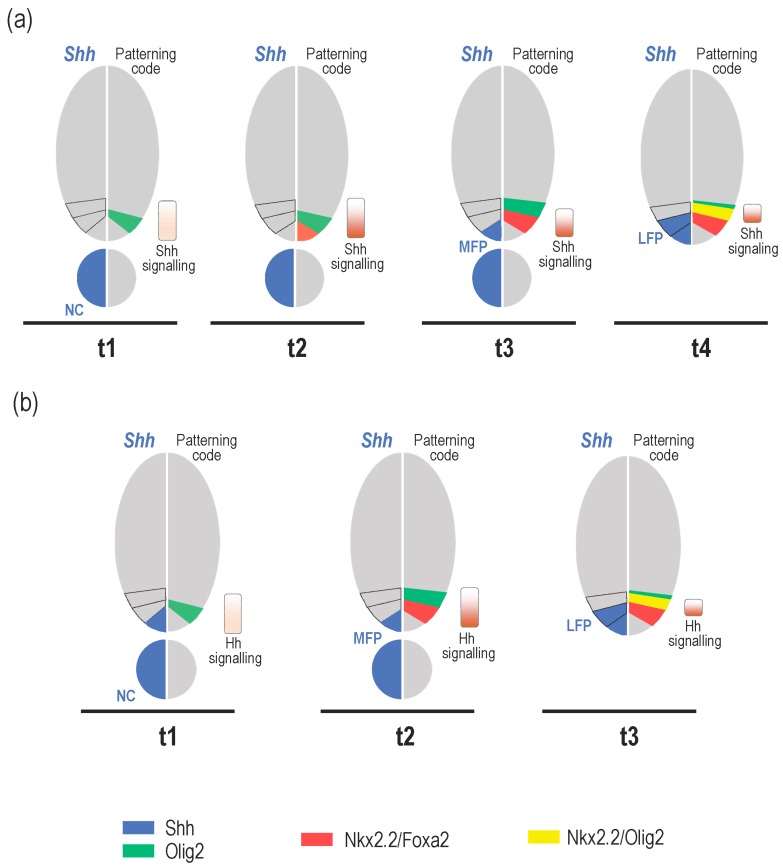
Dynamics of Shh source cells along with neural patterning progression in amniotes (**a**) and zebrafish (**b**). (**a**) Shh, first provided by the notochord (**t1**), induces a low-dose response program in the neural tube revealed by Olig2 expression in ventral progenitor cells. Formation of this pre-pattern is observed over eight days of development (E8) in the mouse embryo [19,26,29]. As development proceeds (**t1–t3**), increasing doses of Shh are progressively provided by the notochord. In response to the resulting activation of Shh signalling in the receiving field, ventral progenitor cells upregulate Nkx2.2 and Foxa2 (**t2**). Expression of these high threshold Shh responsive genes progressively extends dorsally (**t3**) and Olig2 is downregulated in the ventral-most progenitor cells due to the repressive activity of Nkx2.2. In parallel, Shh expression is activated in ventral midline cells (**t3**), thus completing MFP cell differentiation. In these cells, Shh signalling is downregulated and Nkx2.2 expression is turned off. At that time, patterning of neuron-producing domains is achieved. The **t2**–**t3** period of development corresponds roughly to the period between the ages of E1.5-E2 and E8.5-E9.5 in chicks and mice, respectively [19,26,28,29,60]. From that time, MFP becomes the main provider of Shh required to maintain progenitor domains. This is paralleled by a general decrease of Shh signalling activity in ventral progenitor cells while they start generating neurons [20,27]. After a period of several days (**t4**), ventral progenitor cells co-expressing Nkx2.2 and Foxa2 activate Shh expression to form the LFP. This is associated with a decay of Shh signalling in these cells. At that time, Shh signalling is activated in dorsal adjacent progenitor cells that subsequently upregulate Nkx2.2. This change in pattern organization leads to formation of a novel progenitor domain populated by Olig2/Nkx2.2-coexpressing cells that change their fate to generate glial cells. **t4** corresponds to E5.5 in chicks and E11.5 in mice [42,43]. (**b**) At initiation of neural tube patterning in zebrafish (**t1**), Hedgehog ligands are provided by the notochord but also by MFP cells. The time course of Olig2 and Nkx2.2 expression in ventral progenitor cells is conserved. Pattern formation of ventral progenitor domains (**t1**–**t2**) takes place over a short period of time, from 14 to 16 hours post-fertilization (hpf) [43,61,62]. After a period of approximately one day (**t3**), Shh expression expands laterally into the Nkx2.2-expressing domain to form the LFP and the subsequent reorganization of the ventral patterning that is in place from 36 hpf in zebrafish [40,43].

Parallel to these events, important temporal changes in the identity and position of cells that provide the Shh signal are also known to occur both in amniotes and zebrafish, highlighting the dynamic nature of Shh source cells over time (Figure 2). The notochord is the first localised source of Shh that comes in contact with the ventral caudal neural tube as it closes. The ventral medial cells of the neural tube, commonly known as floor plate (FP) cells, have long been recognised to also express Shh. In the past years, the mechanisms that control the development of the FP have received much attention and led to the proposal of a model in which Shh, secreted from the notochord, induces FP formation in the overlying neural tube [47,48]. This model came from experiments performed in amniotes, showing that notochord-derived signals can induce FP differentiation both in vitro and in vivo [49,50,51,52,53]. Conversely, absence of the notochord, resulting from notochord ablation or mutations that disrupt its formation, is accompanied by failure in FP induction [49,51,52,54,55,56]. Further studies rapidly recognised Shh to be the main notochord-provided signal responsible for triggering FP induction [15,16,29,57]. However, whether the notochord-provided Shh is truly responsible for FP cell induction has been a matter of controversy. Specification of FP cells in chicken and zebrafish has been proposed to occur earlier, within the node, independently of the inductive activity of the notochord (for review, see [17,58]). Later in development, a third source of Shh was recognised to form in the ventral-most neural progenitor cells [40,43,59]. These cells, because they differentiate on either side of the floor plate, were named the lateral floor plate (LFP) cells. To avoid confusion of terms, medial cells of the neural tube that are the first to activate Shh will be named the medial floor plate (MFP) instead of FP cells in the following sections. Here, and in an attempt to pave the way for the specific roles for these time-variant Shh sources, we review the temporal dynamics of Shh-source cells and relate these sequential changes to the control of developmental transitions over spinal cord development.

## 2. Specific Functions of the Notochord-Derived Shh

### 2.1. Shh Provided by the Notochord Triggers MFP Differentiation

Although some controversies have arisen concerning the requirement of the notochord-provided Shh for MFP cell induction, recent data confirm its role in controlling MFP differentiation in amniotes. In mouse embryos lacking functional Shh, while initial development of the notochord proceeds properly, MFP cells fail to differentiate [63,64]. The prospective MFP cells are also known to be the direct target of the notochord-derived Shh in mice [65]. For triggering MFP cell differentiation, notochord cells must provide high levels of Shh signalling. This was first recognised from in vitro experiments showing that treatment of neural plate explants with high concentrations of Shh is necessary to trigger the expression of MFP genes [16]. In agreement with these experimental observations, at the time of MFP cell differentiation in vivo, most of the secreted Shh is retained at the surface of notochord cells while these cells are in direct contact with the basal surface of the neural tube [16,18,19,26,29]. Identification of the temporal sequence of genes regulated by Shh in the prospective MFP cells further reinforced this assumption. The earliest reported event occurring in response to the notochord-provided Shh in prospective MFP cells is the concomitant activation of Nkx6.1 and downregulation of Pax6, two transcription factors activated and repressed by high levels of Shh signalling, respectively [4,19,26,66]. Shortly afterwards, Nkx2.2 and Foxa2 (also known as HNF3β), both being direct targets of Shh signalling and also known to respond to high doses of the morphogen, are upregulated in these cells [19,26,28,29,30,31,32,67,68,69,70,71]. This dynamic transcriptional program in prospective MFP cells was supposed to reflect increasing levels of Shh signalling over time, as suggested by in vitro assays showing that elaboration of this transcriptional program indeed depends on increasing Shh concentration thresholds [4,29,66]. Accordingly, in an elegant study based on the direct visualization a GFP tagged version of Shh, Chamberlain and collaborators (2008) were able to show that the level of Shh provided by the notochord increases progressively as ventral medial cells go from expressing Nkx6.1 to upregulating Nkx2.2. At the same time, prospective MFP cells themselves contribute to their sensitization to the Shh signal by expressing the Shh signal enhancers Cdo and Gas1, both required for MFP formation [72,73,74]. Another essential parameter in the control of MFP development is the competence of neural cells to respond to Shh. Grafts of notochord explants initially highlighted that there is only a narrow time window when full differentiation of MFP cells can take place [29,49,59]. Another signal, FGF, was further shown to provide this competence by maintaining transient expression of the transcription factor Nkx2.1 in ventral medial cells of the neural tube [75]. The intersection of FGF signal emanating from the caudal part of the embryo and high threshold Shh signals secreted by the notochord is therefore recognised to provide a spatial and developmental time window that determines generation of MFP cells from ventral medial cells of the neural tube.

At a certain time point, while still in contact with the notochord, prospective MFP cells become refractory to the Shh signalling, a key step in maintaining their identity. If Shh signalling is artificially forced for a long period of time, the prospective MFP cells do not differentiate properly and instead convert their identity to ventral neural progenitors [29,32]. Downregulation of Gas1, an enhancer of the Shh signalling whose expression is negatively regulated by Shh, has been proposed to contribute to the attenuation of Shh signalling in MFP cells [72,73,74]. This attenuation is likely to also rely on the repression of Gli2, the predominant actor in relaying the Shh signal in MFP cells, resulting from Foxa2 expression in these cells [29,76,77,78]. From this time on, the final steps of MFP differentiation are mainly coordinated by Foxa2 that orchestrates induction of the MFP genes Arx and Nato3 and, indirectly, downregulation of Nkx2.2, a cellular context required for activation of Shh expression in MFP cells [29,60,79,80]. Therefore, Foxa2 itself activated by the notochord-provided Shh, is the key factor that relays Shh signalling for full differentiation of MFP cells.

Thus, proper differentiation of the MFP relies on a transient burst of Shh signalling provided by the notochord. After that, Shh continues to be expressed by MFP cells independently of Shh signalling, in agreement with the physical separation of the notochord and MFP cells that occurs at later developmental stages.

### 2.2. Shh Provided by the Notochord Induces Neural Tube Patterning in Amniotes

The role of Shh emanating from the notochord is not limited to formation of the MFP. Notochord-provided Shh is also recognised to play a key role in the elaboration of the p3, pMN and p2 ventral domains [19,25,26,76,81]. In chicks and mice, patterning of the ventral neural tube is initiated even before Shh is expressed in prospective MFP cells [19,25,26,76,81]. In Gli2 mutant mouse embryos lacking MFP cells, production of Shh from the notochord is sufficient to pattern the ventral neural tube, although neural progenitor cells appear to be reduced in number in some domains [76,82]. Likewise, and again despite reduced domain sizes, the neural tube patterning arises in mice, where Shh removal is genetically driven in medial neural cells [25,81]. Importantly, Shh emanating from the notochord is recognised to reach the neural target field by traveling through ventral medial cells as the patterning process is under way [19].

### 2.3. Shh Provided by the Notochord Favours Progenitor Cell Proliferation in Amniotes

Establishment of the neural tube patterning occurs at the same time as the tissue grows [83], a process also regulated by notochord-derived Shh. Notochord removal has long been known to reduce the size of the neural tube [84,85], while implantation of a notochord fragment lateral to the neural tube enhances proliferation of progenitor cells [53,86]. Shh was further recognised to be the notochord signal responsible for causing over-proliferation of neural progenitor cells [87,88,89]. In support of this, mouse embryos depleted for the two endogenous inhibitors of the pathway, Ptc1 and Hip1, exhibit a noticeably enlarged neural tube; this is observed before Shh expression in MFP cells [26].

### 2.4. Role of the Notochord-Provided Shh in Zebrafish

Although one of the first studies involving the notochord-derived Shh in the control of neural cell identity was performed in the zebrafish embryo [61], further analyses revealed an alternative model for MFP cell induction. Notably, MFP develops properly in zebrafish embryos, carrying a mutation in no tail-a (a Brachyury ortholog) and in which notochord cells are absent, thus questioning the involvement of Shh signalling in zebrafish MFP cell induction [90,91]. MFP cells were further shown to form normally in zebrafish embryos homozygous for a deletion of *sonic you*, the zebrafish Shh gene [92,93,94]. However, in contrast to mice and chicks, in which Shh is the only Hedgehog gene expressed by the notochord, in zebrafish, midline mesodermal cells also express *echidna hedgehog*, an Indian hedgehog homologue [95,96]. The third Hedgehog zebrafish gene, *tiggywinkle hedgehog* (twhh), is not expressed in the notochord but is activated together with Shh in MFP cells [97,98]. Yet, proper induction of MFP cells in Shh mutant embryos does not appear to reflect cooperation with the other members of the Hedgehog family since MFP cells differentiate in Shh mutants injected with morpholinos used to knock down twhh [98] or twhh plus ehh [99]. Similarly, mutations in the zebrafish *smoothened* (Smo) or *you-too* (Gli2) genes or treatment with cyclopamine, a Smoothened inhibitor, only partially affect MFP differentiation [62,100,101,102]. Rather, MFP formation is altered in *cyclops* and *one-eyed pinhead* mutants that lack the Nodal-related-2 protein and its receptor, respectively [103,104,105,106,107,108]. Therefore, instead of Shh, the TGFβ signalling factor Nodal has been proposed to be the primary signal responsible for MFP cell specification in zebrafish. Accordingly, expression of Foxa2 (also known as Axial in zebrafish), also required for MFP differentiation in zebrafish, has been proposed to depend on Nodal, instead of Shh [109]. In support of the view that the Hedgehog signalling plays a less prominent role in zebrafish than in amniotes, the two Nkx2.2 paralogs, Nkx2.2a and Nkx2.2b, are never activated in the zebrafish MFP cells [43,62,110]. However, a more recent study showing that Hedgehog signalling, together with Nodal, contributes to induce MFP cells within a short time window spanning from gastrulation to early somitogenesis, challenged the model of Hedgehog-independent induction of MFP cells in zebrafish [29]. Although still discussed for its function in MFP induction, Hedgehog signalling is nevertheless recognised to be required for the maintenance of MFP cell identity in zebrafish. Although zebrafish embryos can form MFP cells in the absence of Hedgehog signalling activity, they indeed prematurely lose expression of MFP markers as development proceeds [100]. Moreover, in *cyclops* and *one-eyed pinhead* mutant embryos, MFP cells finally form in a Shh-dependent manner [107,111]. Thus, both Hedgehog and Nodal contribute to MFP formation in zebrafish. Based on some evidence that Nodal also plays a role in MFP formation in amniotes, the apparent differences in MFP formation between zebrafish and amniotes likely reflect varying contributions of Hedgehog and Nodal signalling to MFP induction and maintenance (for review, see [17] ). Another noticeable aspect of zebrafish MFP formation is that differentiation of these cells and initiation of neural tube patterning do not follow the same temporal sequence as in amniotes. In zebrafish, MFP cell identity, i.e., expression of Shh and Twhh in these cells, is set up prior to establishment of neural tube patterning [12,43,61,62,98,110]. Therefore, in zebrafish, as discussed below, the MFP instead of the notochord is likely the main source of Hedgehog signalling required for formation of ventral neural progenitor domains.

## 3. What Is the Relevance of Forming the MFP as a Secondary Signalling Centre?

### 3.1. Shh Provided by MFP Cells Is Required to Maintain Progenitor Domains in Amniotes

In amniotes, after the establishment of the neural tube patterning, the notochord loses its contact with the neural tube and regresses away from the developing spinal cord as it becomes surrounded by sclerotomal cells. This regression process takes place as neuronal production in the ventral spinal cord is on the way [81]. Therefore, during neurogenesis, the source of Shh is mostly the MFP [19,81]. As mentioned above, Gli2 mutant mouse embryos that fail to develop a MFP are still capable of specifying all primary Shh-dependent ventral neural progenitor populations [76,82]. In these embryos, MNs, while occupying a more ventral position, still differentiate. At first glance, this might reflect that Shh from MFP cells is dispensable for MN differentiation. However, in Gli2 mutant mouse embryos, the notochord does not regress but remains in close contact with the developing spinal cord while it continues to express Shh. Therefore, due to the persistent proximity of the notochord to the ventral spinal cord, it is conceivable that Shh emanating from this tissue might be sufficient to compensate for the loss of MFP cells, precluding any conclusion on the requirement of MFP-derived Shh for induction of neuronal differentiation. A set of more recent studies, however, supports an essential role of the MFP-provided Shh for maintenance of the ventral patterning. Cyclopamine treatment, used in chicken to interrupt Shh at different time windows after establishment of the neural tube patterning, indicated that continued Shh signalling is required to maintain the identities of ventral progenitor cells [25,33,39]. Notably, Olig2 expression is more severely affected than that of Nkx2.2, indicating differential requirement of MFP-provided Shh to maintain the pMN and p3 domains [25,33,39]. Furthermore, mice containing conditional null alleles for Shh were used to test whether an extended period of Shh signalling is also necessary for maintaining the progenitor domains in the mouse neural tube. Deletion of Shh from MFP cells was performed using mice containing a conditional null allele for Shh and transgenes that activate Cre expression in ventral cells only after patterning establishment [25]. In these double transgenic embryos, expression of Shh by the notochord, as well as initial formation of the progenitor domains, are unaffected and, in contrast to what has been observed in embryos lacking Gli2, the notochord separates normally from the neural tube. Similarly to what was observed in chicken, a marked decrease in the number of cells expressing Olig2 and, to a lesser extent, Nkx2.2, was observed, accompanied by a ventral shift in the expression of dorsal markers. Similar observations were more recently reported using a distinct transgenic Cre line that induces genetic deletion of Shh specifically in the MFP, using an MFP-Foxa2-pecific driver [81]. Finally, a reduced number of Olig2-expressing cells was also found in mouse embryos depleted for the MFP transcription factor Arx, in which the levels of Shh expression are left intact in the notochord but markedly reduced in MFP cells [60]. It is therefore assumed that the MFP-derived Shh plays a key role in maintaining the identity of neural progenitor cells after it has been assigned and subsequently the production of the complete set of neurons in the ventral spinal cord. In the chick neural tube, a direct influence of Shh on the control of neural progenitor cell cycle progression and survival has also been evidenced to occur until late stages of development [112]. Therefore, MFP-derived Shh is also likely required to maintain progenitor cell proliferation and prevent cell death over spinal cord development.

### 3.2. MFP-Provided Shh Is Required to Achieve the Neural Tube Patterning in Zebrafish

Spatial patterning of the neural tube is known to be conserved in zebrafish [113] and to also depend on Hedgehog signalling for its establishment [12,40,62,113,114,115,116]. However, the temporality of ventral progenitor domain formation relative to the expression of Hedgehog proteins in the notochord and MFP cells differs compared to amniotes. As mentioned above, zebrafish MFP cells become a source of Hedgehog proteins at earlier stages compared to amniotes, i.e., from mid-gastrula stages [61,98]. At the same time, some patterning genes, including Olig2, Nkx6.1 and Nkx6.2, are already expressed in medial cells of the neural plate [12,62,114]. This therefore opens up the possibility that expression of these genes might be activated by Hedgehog proteins provided by MFP cells, together with or in place of notochord cells. It is worth noting that preventing Hedgehog signalling at early somitogenesis stages, although reducing the levels of Olig2 and Nkx6.1/2 expression, does not totally abolish their expression [12,62,114]. Therefore, in zebrafish, additional signals together with Hedgehog proteins might contribute to initiate patterning of the ventral neural tube. In contrast, elaboration of the p3 and pMN domains requires Hedgehog signalling activity [43,62]. Formation of these two domains is achieved during the segmentation period, by the activation of Nkx2.2a, the Nkx2.2 orthologue, together with downregulation of Olig2 in p3 progenitor cells [43]. The prominent role of the MFP-provided Hedgehog proteins in patterning the p3 and pMN domains in zebrafish was further highlighted by the identification of a novel modulator of Hedgehog signalling activity, named Sulf1 [41,42,43]. Sulf1 is an extracellular sulfatase known to regulate signalling pathway activities by modulating the interaction of ligands with heparan sulphate proteoglycans (HSPGs) at the cell surface [117]. In *Drosophila*, Sulf1 has opposing functions, enhancing Hedgehog release from its source and reducing Hedgehog signalling activity in the responding cells [118]. In the vertebrate spinal cord, Sulf1 only behaves as a positive modulator of Shh signalling [41,42,43]. The enzyme is known to enhance Shh signalling activity in a cell-autonomous manner by stimulating production of active forms of Shh from its source cells [43]. In zebrafish, at the time of patterning establishment, Sulf1 is expressed in MFP cells but not in notochord cells [43,119]. Strikingly, Sulf1 expression in MFP cells follows Hedgehog protein expression but shortly precedes activation of Nkx2.2a in progenitor cells of the p3 domain [43]. Depletion of Sulf1, while not affecting the timing of Olig2 activation, is, however, sufficient to prevent establishment of the p3 domain, a patterning defect accompanied by defective generation of V3 interneurons but normal production of MNs [43]. The characterization of Sulf1, required to activate high-threshold response to Hedgehog, not only places the MFP cells at the core of the patterning establishment in zebrafish but also highlights a novel mechanism of the temporal control of the Hedgehog signalling, based on the temporal evolution of ligand source identity.

## 4. Role of Shh Provided by Lateral Floor Plate (LFP) Cells

### 4.1. Formation and Identity of LFP Cells

The existence of two distinct types of FP cells was initially proposed following observation of differences in the spatial expression of FP molecular markers. In the chick and zebrafish neural tube, Foxa2 and other floor plate markers such as Netrin-1 or Fkd4, were shown to display a broader domain of expression than that of Shh [15,59,94,120,121,122,123]. The inner cells were then named MFP cells, while cells located on either side and displaying a partial floor plate character were named LFP cells. Therefore, LFP cells were so called in the literature before being identified as prospective Shh-producing cells [15,94,122,123]. It was only after the observation that these cells activate the expression of Shh in chicken and zebrafish that the LFP was considered to be a secondary source of the morphogen in the developing spinal cord [40,43,59]. Initiation of Shh expression in LFP cells is a late event that occurs long after establishment of the neural tube patterning and onset of neuronal production in chicks and zebrafish [40,43,59] but also in mice (Ohayon, D., Escalas, N., Danesin, C. and Soula C., CBD, University of Toulouse, France. Unpublished work, 2017). At the same time, induction of this secondary source of Shh was recognised to occur within the Nkx2.2-expressing p3 progenitor domain that earlier on generated V3 ventral interneurons [43,59,121]. Therefore, ventral cells of the neural tube, initially named LFP cells because they express the floor plate marker Foxa2 but not Shh, are nothing more than neural progenitor cells of the p3 domain. To avoid any confusion, in the following sections the terminology LFP is used for p3 cells only from the time these cells gain expression of Shh. On the basis of this definition, LFP cells are characterised by the expression of a set of markers also expressed in MFP cells, including Foxa2 and Shh, as previously mentioned, but also Sulf1 [43,124]. As reported for MFP cells [20,29], LFP cells rapidly become refractory to Shh as they differentiate [42,43]. However, in contrast to MFP cells that must downregulate Nkx2.2 to achieve their differentiation program [29], LFP cells maintain Nkx2.2 expression [43,59]. Of note, this is far from being at odds with decay of Shh signalling in LFP cells since maintenance of Nkx2.2 expression is known to no longer depend on Shh at the time of LFP induction [33,39,43]. As mentioned above, Foxa2, expressed at a high level in MFP prospective cells, is also activated but at a lower level in ventral neural progenitor cells of the prospective p3 domain [28,29]. This transcription factor, known to be necessary and sufficient to induce Shh expression [29,67,69], remains expressed until the time of LFP induction, suggesting that it might also be involved in Shh upregulation in prospective LFP cells of the p3 domain [43,59]. However, the long period between the onset of Foxa2 expression and Shh upregulation in these cells suggest that additional factors might be required for LFP differentiation. Although the timing of LFP differentiation has been precisely defined in the chick and zebrafish, the mechanisms responsible for induction of this secondary source of Shh are largely unknown. Although grafts of notochord or MFP cells close to the neural tube are able to induce ectopic formation of LFP cells, the long period of time required to obtain a full LFP character (3–5 days) precludes coming to a conclusion as to the direct effect of Shh signalling [59]. Whether differentiation of LFP cells depends on Shh, which is still provided by MFP cells at the time of LFP formation, therefore remains an open question.

### 4.2. Is There a Specific Function for LFP-Derived Shh?

A set of conclusive arguments favouring a decisive role for LFP-derived Shh in the control of gliogenesis has recently emerged. Such a link was first proposed when it was realised that the formation of this secondary source of Shh is temporally correlated with the induction of oligodendrocyte precursor cells (OPC) from progenitor cells of the pMN domain [43]. Shh signalling being still required at these late stages for induction of OPCs [37,38,39,40,43], it was tempting to think that formation of this novel source of Shh might represent a definitive signal for triggering the neuroglial switch. Accordingly, a temporal rise in Shh signalling activity, assessed by upregulation of the Shh responsive genes Ptc1 and Gli1 in progenitor cells of the pMN domain, was further shown to occur at the onset of OPC generation [41,42,43]. Thus, after a decline of Shh signalling during the neurogenic period [20,27], Shh activity appears to peak again long after the establishment of the neural patterning (from hours in zebrafish to days in amniotes). In agreement, the high-threshold, Shh-responsive gene Nkx2.2 is at that time activated in Olig2-expressing progenitor cells, leading to formation of a new domain (p* domain) populated by cells co-expressing Nkx2.2 and Olig2 [39,42,43,44,45,46]. Therefore, LFP differentiation, by bringing a source of Shh into closer proximity to Olig2 progenitor cells, likely contributes to creating the burst of Shh required to change the fate of pMN progenitor cells, from producing MNs to generating OPCs. However, at the time of patterning rearrangement, Shh is still expressed in MFP cells [42,43,59]. Therefore, to assign this role specifically to LFP-derived Shh, one must be able to prevent expression of Shh in LFP cells without affecting it in MFP cells. Although this has not been directly tested, work performed to unravel the role of Sulf1 in OPC induction further supports the view that Shh provided by MFP cells is dispensable for formation of the p* domain and thereby specification of OPC [42,43]. Sulf1, whose expression is restricted to MFP cells over the period of neuronal production, is upregulated in LFP cells just after their formation [43]. Experiments performed in chick embryo showed that downregulating Sulf1, specifically in the LFP, is sufficient to impair OPC specification, whereas these cells develop properly when knockdown of Sulf1 is limited to the MFP [42]. Although further work is needed to ascertain that Shh provided by the MFP is not required for triggering the MN to OPC fate switch, these observations indicate that the LFP-derived Shh plays a prime role in controlling the temporal fate change of at least a subset of progenitor cells.

## 5. Conclusions

In the developing neural tube, differential cellular responses to Shh are known to be mediated by the competence of the receiving cells that change with time, by the dose of the morphogen provided to the receiving cells but also by the duration of the signal. These complex processes are paralleled by temporal dynamics of Shh source cells. The notochord is the first localised source of Shh; then the source moves to the MFP and, finally, an additional source, the LFP, is added. Prevalent roles for each source can be proposed at different developmental periods. The notochord should be considered the essential actor of the neuronal cell diversification in the ventral neural tube. The MFP should be seen as the main guarantor of maintenance of the work done by the notochord. Finally, the LFP, by changing the fate of neural progenitor cells, should be recognised as an additional provider of cell diversification in the developing spinal cord. What could be the relative input of moving the Shh sources over time? Moving the morphogen source changes the position of the receiving cells relative to the source and is also expected to increase the morphogen levels in front of it. The amplitude of the Shh gradient is known to change as the ventral neural tube is patterned [19,20] and the successive formations of the MFP and, later on, LFP are likely to contribute to generating cell diversity by changing the spatial profile of the Shh gradient. Lastly, while MFP formation is known to come from the intersection of FGF signalling and the Shh gradient itself [75], the mechanism underlying LFP induction has yet to be determined. Distinct neuronal populations, already differentiated at the time of LFP induction, are known to become providers of FGFs [3]. It is therefore tempting to speculate that prospective LFP cells (p3 domain) that share many aspects with prospective MFP cells in terms of molecular identity experience the same intersection of FGF and Shh signalling as the prospective MFP cells at earlier developmental stages. Understanding how moving the Shh source integrates into the spatiotemporal hierarchy of inductive events that allow for the generation of neural cell diversity remains a future challenge.

## Abbreviations

Shh: Sonic Hedgehog, Hh: Hedgehog, NC: notochord; FP: floor plate; MFP: medial floor plate; LFP: lateral floor plate; MN: motor neuron; OPC: oligodendrocyte precursor cell.
